# Longitudinal peripheral immune dynamics during anti-programmed cell death 1 neoadjuvant therapy in esophageal squamous cell carcinoma

**DOI:** 10.3389/fimmu.2026.1917487

**Published:** 2026-10-12

**Authors:** Linpeng Li, Lingwen Meng, Xinru Chen, Jiahui Li, Pengfei Li, Bo Dong, Chengjing Huang, Wei Zhou, Nuan Li, Guodong Sun, Jiawei Li, Guojie Tan, Li Jiang, Dan Tian

**Affiliations:** 1 School of Life Science and Technology, Jinan University, Guangzhou, China; 2 Institute of Biomedical Translational Research, Jinan University, Guangzhou, China; 3 The Affiliated Guangdong Second Provincial General Hospital of Jinan University, Guangzhou, China; 4 Department of Oncology, Integrated Hospital of Traditional Chinese and Western Medicine, Southern Medical University, Guangzhou, China; 5 Department of Otorhinolaryngology Head and Neck Surgery, Guangzhou Twelfth People’s Hospital (The Affiliated Twelfth People’s Hospital of Guangzhou Medical University), Guangzhou Medical University, Guangzhou, China; 6 Guangzhou Purui Biotechnology Co., Ltd, Guangzhou, China; 7 Xinzhou District Maternal and Child Health Hospital, Wuhan, China; 8 Guangdong Provincial Key Laboratory of Spine and Spinal Cord Reconstruction, The Fifth Affiliated Hospital of Jinan University, Heyuan, China; 9 Guangzhou Twelfth People’s Hospital (The Affiliated Twelfth People’s Hospital of Guangzhou Medical University), Guangzhou Medical University, Guangzhou, China; 10 Department of VIP Inpatient and Department of Medical Oncology, State Key Laboratory of Oncology in South China, Sun Yat-sen University Cancer Center, Guangzhou, China; 11 Department of Thoracic Surgery, Guangdong Provincial People’s Hospital, Guangdong Academy of Medical Sciences, Southern Medical University, Guangzhou, China

**Keywords:** anti-PD-1, esophageal squamous cell carcinoma, gamma-delta T cells, neoadjuvant therapy, peripheral immunophenotyping

## Abstract

**Introduction:**

Anti-programmed cell death 1 (PD-1)-based neoadjuvant therapy is increasingly used for esophageal squamous cell carcinoma (ESCC), but paired peripheral immune dynamics and their exploratory relationship to response remain incompletely characterized.

**Methods:**

We performed a retrospective longitudinal flow-cytometry study of 39 patients with ESCC treated with anti-PD-1-based neoadjuvant therapy, including 20 patients with paired pre-treatment and post-treatment/preoperative peripheral immune profiles. The primary paired analysis tested 75 marker-defined immune features using Wilcoxon signed-rank tests with Benjamini-Hochberg false discovery rate (FDR) correction. Exploratory extensions included response-associated changes in candidate immune features, clinical burden context, baseline-dependence, and public tissue-context bioinformatics. Public tissue data were used only as descriptive tissue context.

**Results:**

Four features reached FDR q<0.05 in the primary paired screen: PD-1^+^ CD8 T cells, PD-1^+^ Vδ1 γδ T cells, PD-1^+^ Vδ2 γδ T cells, and CD19^+^ B cells. PD-1-defined features were interpreted as assay-defined detectable PD-1 staining readouts because therapeutic anti-PD-1 receptor occupancy or epitope masking may affect post-treatment staining. CD19^+^ B cells remained the non-PD-1 q<0.05 anchor in the PD-1-excluded paired sensitivity screen. In the pathology-evaluable paired cohort (n=17), no response-stratified candidate reached q<0.05; the change in NKG2D^+^ natural killer (NK) cells was the strongest nominal exploratory and hypothesis-generating signal.

**Discussion:**

These data support treatment-associated marker-defined peripheral immune dynamics and generate response-linked hypotheses requiring prospective validation.

## 1 Introduction

ESCC remains a major global health problem, with marked geographic heterogeneity and a substantial mortality burden (1–3). For locally advanced and resectable disease, neoadjuvant multimodality treatment is an established component of curative-intent management (4, 5). Immune checkpoint blockade has expanded this therapeutic landscape: adjuvant nivolumab improved disease-free survival after neoadjuvant chemoradiotherapy and surgery in esophageal or gastroesophageal junction cancer, and multiple neoadjuvant anti-PD-1-based studies in ESCC have reported meaningful pathological responses when checkpoint blockade is combined with chemotherapy or chemoradiotherapy (6–13).

Despite this clinical progress, the peripheral immune dynamics accompanying anti-PD-1-based neoadjuvant therapy have not been fully elucidated. Longitudinal blood immunophenotyping can capture within-patient changes across T-cell, gamma-delta(γδ) T-cell, NK-cell, and B-cell compartments, complementing tissue and pathology analyses while reducing some between-patient heterogeneity (14–18). Peripheral immune monitoring has also been informative in immune-checkpoint-treated solid tumors, although such findings require cohort-specific interpretation (19–21). Flow cytometry is well suited for this purpose, but marker-defined populations should not be equated with cellular function without orthogonal evidence. For example, PD-1, CD57, NKG2D, NKp30, NKp46, CD21, CD24, CD27, and CD38 define assay readouts and population gates; they do not by themselves establish exhaustion, antigen specificity, cytotoxicity, suppressive activity, or causal antitumor function (22, 23).

PD-1 staining requires particular caution in an anti-PD-1-treated cohort. Therapeutic antibodies may occupy PD-1 or mask detection antibody epitopes, causing post-treatment flow cytometric PD-1 signals to reflect detectable/free epitope availability rather than total PD-1 receptor abundance (24–31). Therefore, paired decreases in PD-1-defined populations after therapy require precise terminology and should not be interpreted as direct evidence of reduced total PD-1 expression or altered T-cell state unless clonal interference or receptor-occupancy testing is available.

Here, we analyzed a retrospective ESCC flow-cytometry cohort treated with anti-PD-1-based neoadjuvant therapy. The study was organized around longitudinal peripheral immune pharmacodynamics: first, a paired 75-phenotype treatment screen; second, a prespecified pathology-anchored analysis of immune deltas; and third, independent public ESCC tissue transcriptomics. The central aims were to characterize treatment-associated peripheral immune remodeling, determine whether selected immune changes differed by postoperative pathology, examine continuous disease-burden associations, and place the peripheral findings within tissue immune programs.

## 2 Materials and methods

### 2.1 Study design and cohort

This retrospective translational study used deidentified derived clinical, pathology, and peripheral blood flow-cytometry tables from patients with ESCC treated with anti-PD-1-based neoadjuvant therapy. The immune-flow cohort included 39 patients. The primary paired immune cohort included 20 patients with both pre-treatment and post-treatment/preoperative peripheral blood flow cytometry. The pathology-evaluable paired cohort included 17 patients: 9 had MPR, including 3 with pCR, and 8 had non-MPR. Denominator-specific reporting was used throughout; cohort-level fields, paired immune analyses, and pathology-stratified analyses were not merged across incompatible denominators. Among the 19 patients who lacked paired data, 15 did not return to the hospital for subsequent examinations and treatment, and 4 explicitly refused the post-treatment blood draw. Within the paired cohort of 20 patients, 17 underwent scheduled surgical treatment and were therefore evaluable for pathological response.

### 2.2 Treatment, sampling, and endpoint context

All 39 patients received anti-PD-1-based neoadjuvant chemotherapy. The anti-PD-1 agents were tislelizumab (n=18), sintilimab (n=8), camrelizumab (n=6), nivolumab (n=5), and toripalimab (n=2). After harmonization of synonymous registry terminology, the chemotherapy backbone was albumin-bound paclitaxel plus carboplatin in 35 patients and liposomal paclitaxel plus lobaplatin in 3 patients; the detailed chemotherapy entry was unavailable for 1 patient. Sixteen patients received 2 treatment cycles and 23 received 3 cycles. For the paired cohort, post-treatment blood was collected approximately 3–4 weeks after the final treatment, and surgery was performed within 1 week after the post-treatment blood draw. The median interval between paired blood collections was 91.2 days [interquartile range (IQR), 79.5-107.4; range, 42.0-143.0]. No patient received preoperative radiotherapy; eight registry radiotherapy records referred to postoperative treatment outside the analyzed interval. Peripheral blood was collected from April 2023 to May 2024 in sodium-heparin tubes and processed within 12 hours. MPR was defined as 10% or less residual viable tumor, and pCR was defined as no residual viable tumor in the resected primary tumor and sampled regional lymph nodes (ypT0N0) (32–34). Registry/radiographic response was summarized as CR/PR versus SD/PD when available. Continuous clinical-burden variables included residual tumor size and the pathologic lymph-node-positive ratio, calculated as the number of positive lymph nodes divided by the total number of examined lymph nodes, when available.

### 2.3 Peripheral blood processing and flow cytometry

Available workflow records support a whole-blood staining and red-cell lysis workflow. Package-specific antibodies were retrieved from refrigerated storage, checked against the panel, vortexed, briefly centrifuged, and 10 uL antibody was added to each flow tube. All antibodies were used at a working dilution of 1:200. Whole blood was mixed by gentle inversion, and 100 uL blood was added using reverse pipetting. Tubes were vortexed and incubated protected from light for 15 minutes. Red-cell lysis was performed using 1 mL room-temperature lysing solution, followed by protected-from-light lysis for 15 minutes. Tubes were centrifuged, washed with PBS, centrifuged again, decanted, resuspended in 200 uL PBS, and acquired according to the documented workflow.

Flow cytometry was performed on a BD FACSLyric instrument, and FlowJo v10.1 was used for analysis. Debris handling used FSC/SSC, and doublet handling used FSC-A/FSC-H. Given the retrospective nature of this clinical cohort, fluorescence-minus-one (FMO) and isotype controls were not routinely collected. Consequently, marker-positive gates were robustly established using internal negative control cell populations (e.g., non-T cells for T cell markers), consistent with standard practices for retrospective clinical flow cytometry. Compensation used single-stained compensation tubes and was manually reviewed or adjusted in FlowJo. Acquisition targeted 100,000 lymphocyte events per sample; no subset-specific minimum event-count rule was claimed. Acquisition and gating/review personnel were unaware of clinical response or group status. Flow-cytometry reporting and interpretation were aligned with standard immunophenotyping and flow-cytometry reporting principles where source records allowed (14–18).

### 2.4 Antibody panel, gating, and marker-defined phenotypes

The antibody panel is provided in **Supplementary Table S6A**. Clone, supplier/vendor, and catalog number were retained from author-documented antibody records. RRID and lot fields are marked unavailable/not verifiable where source records were not available. Marker-defined phenotype definitions and parent denominators are provided in **Supplementary Table S6B**. Detailed gating strategies and representative plots are shown in **Supplementary Figure S1**. Event counts for low-frequency immune subsets are provided in **Supplementary Table S7**. Fresh whole blood was stained directly using a standard red-cell lysis workflow. Viability dyes and intracellular fixation/permeabilization steps were not utilized; intact lymphocytes were gated based on standard forward and side scatter properties.

### 2.5 PD-1 staining readout caveat

PD-1-defined flow features were treated as assay-defined detectable PD-1 staining readouts after anti-PD-1 exposure. No study-specific clone-interference or receptor-occupancy assay testing was performed. Therapeutic antibody receptor occupancy, epitope masking, and clone-specific staining behavior may affect post-treatment PD-1 detection (24–31); therefore, post-treatment PD-1 staining results should not be interpreted as total PD-1 expression or T-cell state.

### 2.6 Public ESCC tissue transcriptomic contextualization

The independent public single-cell RNA sequencing (scRNA-seq) ESCC dataset OMIX005710 (35) comprised 46 tissue samples from 22 patients. Raw single-cell gene counts were aggregated once per tissue sample to generate pseudobulk transcriptomic profiles. Duplicate gene symbols were summed, low-expression genes were filtered with edgeR, trimmed mean of M-values normalization was applied, and limma-voom moderated t statistics were used for preranked gene-set enrichment analysis (GSEA). The running enrichment score (**Figure 1B**) was calculated after ranking all genes in descending order according to the limma-voom moderated t statistic for the post-NAT versus pre-NAT tumor contrast. Moving down the ranked list, the score increased when a gene belonging to the tested program was encountered (weighted by the absolute moderated t statistic) and decreased by an equal amount when a non-member gene was encountered. The enrichment score was defined as the largest signed deviation of this running score from zero. The normalized enrichment score (NES) was then obtained by normalizing the enrichment score against the gene-set-specific null distribution generated by fgseaMultilevel.

**Figure 1:**
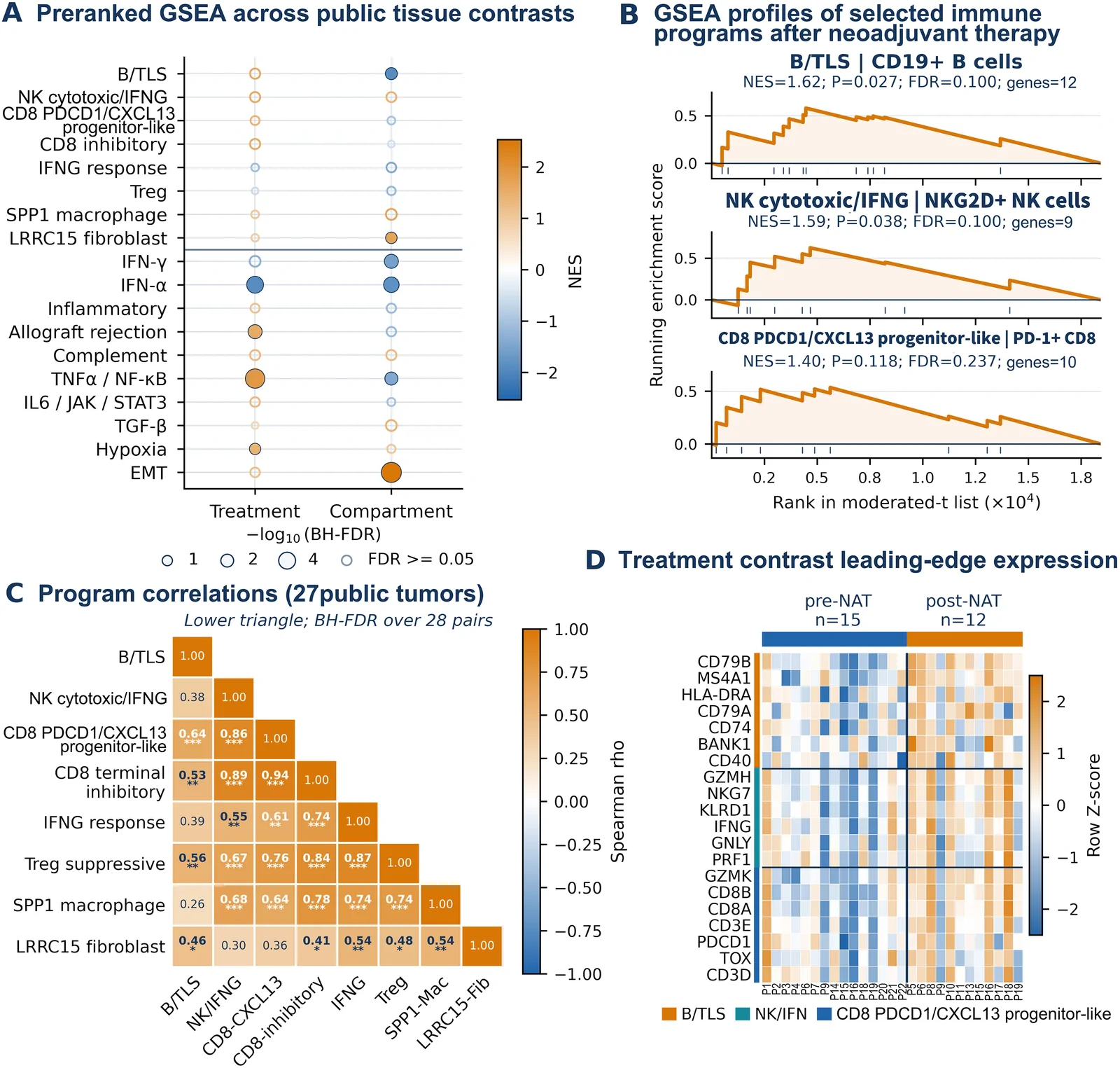
Public ESCC tissue transcriptomics provides biological context for peripheral immune remodeling. **(A)** Preranked gene-set enrichment analysis (GSEA) across public tissue contrasts in the independent OMIX005710 cohort. The treatment contrast compares post-neoadjuvant-therapy (post-NAT, n=12) with pre-NAT tumors (n=15); the compartment contrast compares paired post-NAT tumor and adjacent tissues (n=11). Eight prespecified tumor-microenvironment programs and ten selected Hallmark pathways are displayed. **(B)** GSEA enrichment plots for prespecified tissue programs, explicitly mapped to their corresponding peripheral immune candidates. Normalized enrichment scores (NES), P-values, and false-discovery rates (FDR) are embedded directly within each plot. **(C)** Spearman correlations among the eight prespecified programs across 27 public tumors, with BH-FDR correction across 28 unique program pairs. **(D)** Row-standardized leading-edge gene expression (Z-score) for the three selected programs in panel **(B)** Columns are ordered by treatment group and patient ID without sample clustering. Color values were clipped at ±2.5 for display purposes. ESCC, esophageal squamous cell carcinoma; GSEA, gene-set enrichment analysis; TLS, tertiary lymphoid structure; NK, natural killer; IFN, interferon.

The treatment contrast compared post-neoadjuvant-therapy (post-NAT) tumors (n=12) with pre-NAT tumors (n=15), with patient ID included as a block; six patients with paired pre/post tumor samples were analyzed separately as a sensitivity analysis. The compartment contrast compared paired post-NAT tumor and adjacent tissues from 11 patients. Although 12 post-NAT tumor samples and 12 post-NAT adjacent samples were available overall, 11 patients had both tissues and therefore contributed to the paired compartment contrast. GSEA evaluated eight prespecified tumor-microenvironment programs and ten biologically selected MSigDB Hallmark pathways, with BH-FDR controlled separately by gene-set family within each contrast. Spearman correlations among the eight prespecified programs were calculated across 27 tumor samples with BH-FDR correction across 28 unique program pairs. For leading-edge expression visualization (**Figure 1D**), the row Z-score was calculated separately for each gene across the 27 tumor samples. For gene g in sample s, Zgs = (Egs − mean[Eg])/SD[Eg], where Egs is the TMM-voom log2 counts-per-million value. Thus, a Z-score of 0 represents the mean expression of that gene across the 27 samples, whereas +1 and −1 represent one standard deviation above and below the gene-specific mean, respectively. Values were clipped at ±2.5 for color display only. Internal response and pathology labels were not used in the public-data analyses. Analyses were performed in R 4.6.1 with edgeR 4.10.1, limma 3.68.4, fgsea 1.38.0, msigdbr 26.1.0, and MSigDB 2026.1.Hs; fgseaMultilevel used a fixed random seed of 20260730.

### 2.7 Statistical analysis

The primary paired analysis used paired Wilcoxon signed-rank tests across 75 marker-defined immune features with Benjamini-Hochberg false-discovery-rate correction across the full primary feature family (36, 37). A PD-1-excluded sensitivity screen repeated the paired-screen framework after excluding features with PD-1/CD279/PDCD1 terms. Exploratory response candidate analyses used a predefined 12-feature family across baseline, post-treatment, and post-pre delta layers. Group comparisons used Mann-Whitney tests, group medians, median differences, Cliff’s delta, and BH-FDR adjustment within the predefined family and layer (38, 39). Spearman correlations were used for lymph-node-positive ratio burden context and baseline-dependence checks. PD-L1 combined positive score and residual tumor size were summarized descriptively. No threshold-based or performance-model analysis was constructed. Observational and marker-reporting principles informed the conservative interpretation of exploratory results (40, 41).

### 2.8 Ethics and author statements

The study protocol was reviewed and approved by the Ethics Review Committee of Guangdong Provincial People’s Hospital (approval No. KY-Z-2022-081-01; approval date: May 26, 2022). All patients provided written informed consent. The clinical and flow-cytometry data analyzed in this study are not publicly available because of ethics and patient-privacy restrictions. Deidentified aggregate tables supporting the main findings may be made available from the corresponding author upon reasonable request and subject to institutional and ethics approvals, where applicable.

## 3 Results

### 3.1 Cohort, treatment context, and endpoint availability

The immune-flow cohort included 39 patients: 20 with paired pre-treatment and post-treatment/preoperative immune profiles and 19 with baseline profiles only. Postoperative pathology was available for 18 of 39 patients in the full immune cohort and for 17 of 20 patients in the paired cohort. The pathology-evaluable paired cohort comprised 9 patients with MPR, including 3 with pCR, and 8 with non-MPR. Registry/radiographic response was available for all paired patients (CR/PR, n=15; SD/PD, n=5). The lymph-node-positive ratio, PD-L1 combined positive score, and residual tumor diameter were available for 16, 10, and 12 paired patients, respectively.

**Figure 2** presents the cohort flow and the derivation of the analysis populations. The detailed clinical characteristics, treatment parameters, and denominator-specific data availability are summarized in **Table 1**.

**Figure 2:**
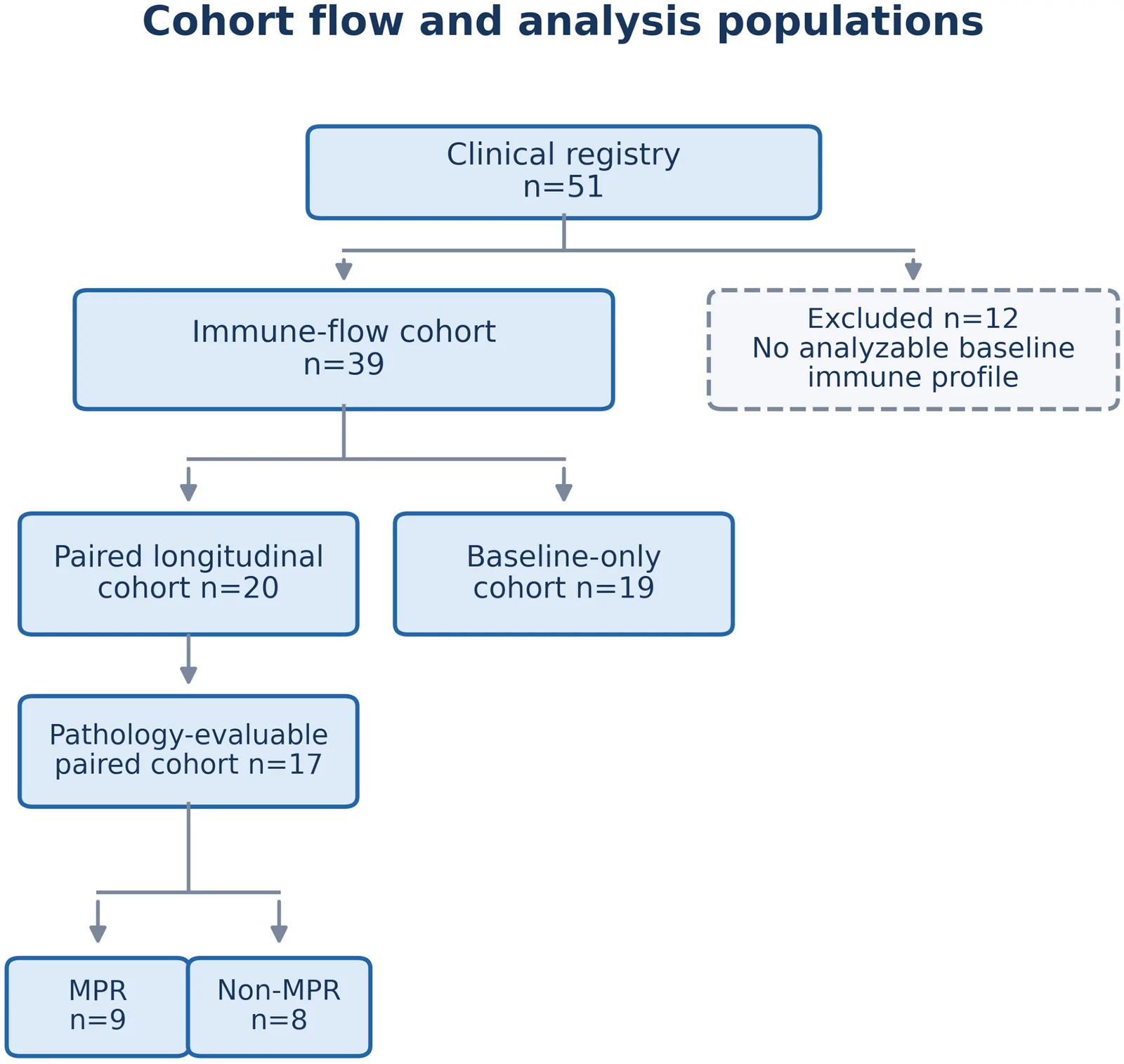
Study cohort flow and analysis populations. The flowchart details patient exclusion and subdivision into the paired longitudinal and baseline-only cohorts. Three of the 9 MPR patients achieved pathologic complete response (pCR). Detailed blood-sampling timelines and data availability are provided in **Table 1**. MPR, major pathologic response (defined as ≤10% residual viable tumor); pCR, pathologic complete response.

**Table 1:** Clinical characteristics of the patient cohorts.

Characteristic	Paired immune cohort	Pathology-evaluable paired cohort	MPR (including pCR)	Non-MPR
Total, n	20	17	9	8
Age, median [IQR]	65.5 [58, 68.2]	65 [58, 66]	66 [60, 66]	64 [53.8, 65.8]
Female sex, n/N (%)	3/20 (15.0%)	3/17 (17.6%)	1/9 (11.1%)	2/8 (25.0%)
Clinical T category, n/N (%)	cT2 2/20 (10.0%); cT3 15/20 (75.0%); cT4 3/20 (15.0%)	cT2 2/17 (11.8%); cT3 12/17 (70.6%); cT4 3/17 (17.6%)	cT2 1/9 (11.1%); cT3 7/9 (77.8%); cT4 1/9 (11.1%)	cT2 1/8 (12.5%); cT3 5/8 (62.5%); cT4 2/8 (25.0%)
Clinical N category, n/N (%)	cN0 5/20 (25.0%); cN1 7/20 (35.0%); cN2 7/20 (35.0%); cN3 1/20 (5.0%)	cN0 5/17 (29.4%); cN1 6/17 (35.3%); cN2 5/17 (29.4%); cN3 1/17 (5.9%)	cN0 4/9 (44.4%); cN1 3/9 (33.3%); cN2 2/9 (22.2%); cN3 0/9 (0.0%)	cN0 1/8 (12.5%); cN1 3/8 (37.5%); cN2 3/8 (37.5%); cN3 1/8 (12.5%)
Anti-PD-1 agent, n/N (%)	Tislelizumab 10/20 (50.0%); Sintilimab 5/20 (25.0%); Camrelizumab 3/20 (15.0%); Nivolumab 1/20 (5.0%); Toripalimab 1/20 (5.0%)	Tislelizumab 9/17 (52.9%); Sintilimab 3/17 (17.6%); Camrelizumab 3/17 (17.6%); Nivolumab 1/17 (5.9%); Toripalimab 1/17 (5.9%)	Tislelizumab 5/9 (55.6%); Sintilimab 2/9 (22.2%); Camrelizumab 1/9 (11.1%); Nivolumab 0/9 (0.0%); Toripalimab 1/9 (11.1%)	Tislelizumab 4/8 (50.0%); Sintilimab 1/8 (12.5%); Camrelizumab 2/8 (25.0%); Nivolumab 1/8 (12.5%); Toripalimab 0/8 (0.0%)
Chemotherapy backbone, n/N (%)	Albumin-bound paclitaxel plus carboplatin: 19/20 (95.0%) Liposomal paclitaxel plus lobaplatin: 1/20 (5.0%)	Albumin-bound paclitaxel plus carboplatin: 16/17 (94.1%) Liposomal paclitaxel plus lobaplatin: 1/17 (5.9%)	Albumin-bound paclitaxel plus carboplatin: 9/9 (100.0%)	Albumin-bound paclitaxel plus carboplatin: 7/8 (87.5%) Liposomal paclitaxel plus lobaplatin: 1/8 (12.5%)
Postoperative pathology evaluable, n/N (%)	17/20 (85.0%)	17/17 (100.0%)	9/9 (100.0%)	8/8 (100.0%)
MPR among pathology-evaluable patients, n/N (%)	9/20 (45.0%)	9/17 (52.9%)	9/9 (100.0%)	0/8 (0.0%)
pCR among pathology-evaluable patients, n/N (%)	3/20 (15.0%)	3/17 (17.6%)	3/9 (33.3%)	0/8 (0.0%)
Registry/radiographic CR/PR, n/N (%)	15/20 (75.0%)	12/17 (70.6%)	8/9 (88.9%)	4/8 (50.0%)
Registry/radiographic SD/PD, n/N (%)	5/20 (25.0%)	5/17 (29.4%)	1/9 (11.1%)	4/8 (50.0%)
Pathologic LN positive ratio available, n/N (%)	16/20 (80.0%)	16/17 (94.1%)	9/9 (100.0%)	7/8 (87.5%)
Residual tumor size available, n/N (%)	12/20 (60.0%)	12/17 (70.6%)	6/9 (66.7%)	6/8 (75.0%)
PD-L1 CPS available, n/N (%)	10/20 (50.0%)	10/17 (58.8%)	2/9 (22.2%)	8/8 (100.0%)

Values are n/N (%) unless otherwise indicated. Registry/radiographic response and postoperative pathologic response are distinct endpoints. Pathology-evaluable denominators are used for MPR and pCR; pCR is a subset of MPR. MPR was defined as 10% or less residual viable tumor, and pCR was defined as no residual viable tumor in the resected primary tumor and sampled regional lymph nodes (ypT0N0). The pathologic LN-positive ratio was calculated as positive lymph nodes divided by examined lymph nodes. Chemotherapy terminology was harmonized to the final clinician-confirmed backbone categories. CPS, combined positive score; CR, complete response; IQR, interquartile range; LN, lymph node; MPR, major pathologic response; NAT, neoadjuvant therapy; pCR, pathologic complete response; PD, progressive disease; PD-1, programmed cell death protein 1; PR, partial response; SD, stable disease.

### 3.2 Treatment-associated peripheral immune dynamics

The primary paired 75-feature analysis identified four marker-defined features with BH-FDR q<0.05: PD-1+ CD8 T cells (delta median -18.7, effect size -0.99, q=1.4e-04), PD-1+ Vδ1 γδ T cells (delta median -17.8, effect size -1.00, q=1.4e-04), CD19+ B cells (delta median -3.25, effect size -0.962, q=3.3e-04), and PD-1+ Vδ2 γδ T cells (delta median -3.69, effect size -0.857, q=0.005). **Figure 3** displays the full 75-feature paired screen, patient-level paired trajectories for the four FDR-supported features, major-lineage deltas, and the PD-1-excluded sensitivity landscape. Comprehensive statistical summaries are integrated directly within **Figure 3B**. **Supplementary Table S1** provides the full 75-feature paired analysis. **Table 2** provides concise numerical summaries for the four FDR-supported treatment-associated features.

**Figure 3:**
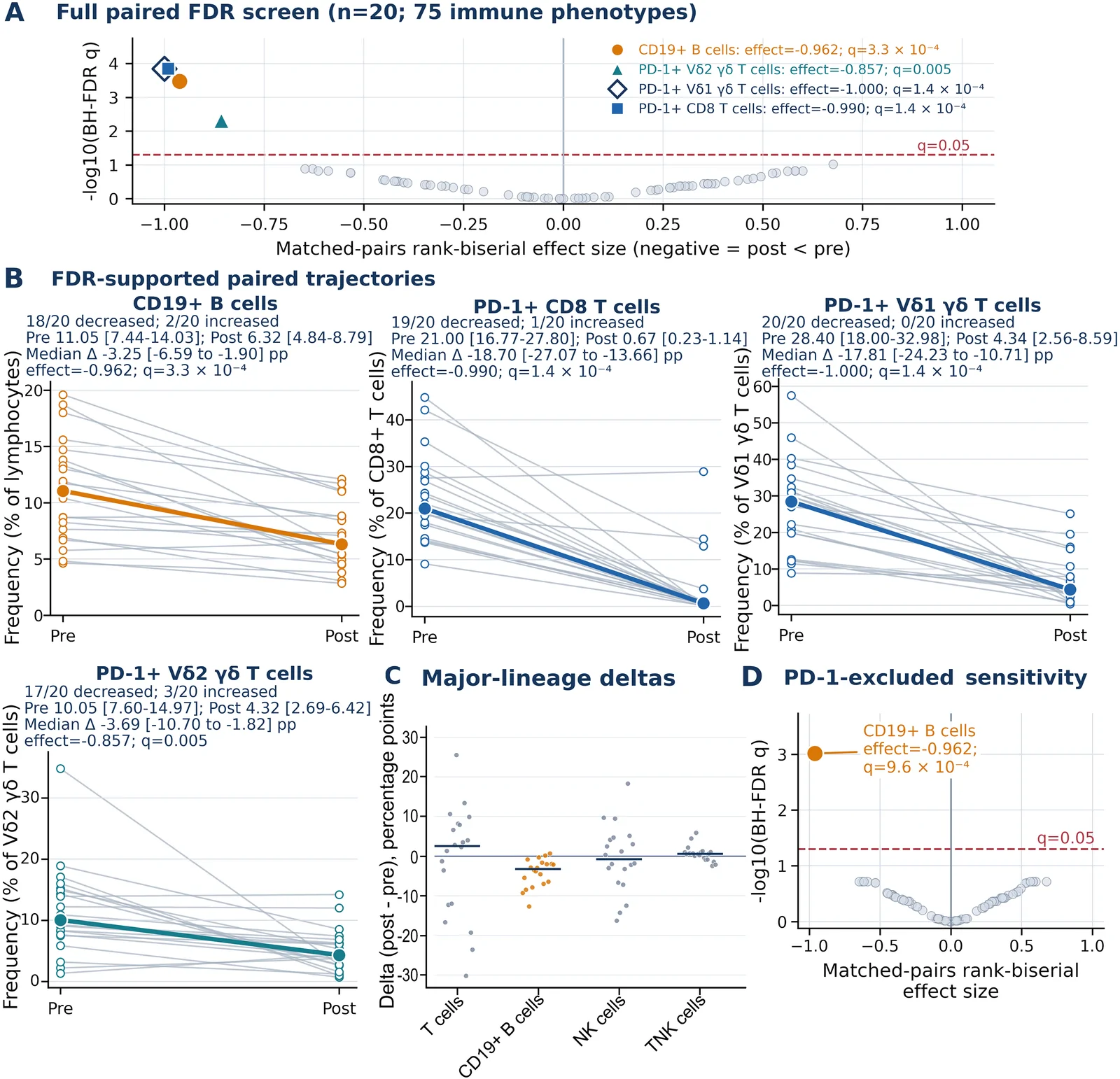
Treatment-associated peripheral immune dynamics. Delta denotes post-treatment minus pre-treatment frequencies in percentage points (pp) for the paired cohort (n=20). **(A)** Full 75-phenotype paired screen. The dashed line denotes Benjamini-Hochberg false-discovery-rate (BH-FDR) q=0.05 (two-sided Wilcoxon signed-rank tests). **(B)** FDR-supported patient-level paired trajectories. Comprehensive statistical summaries are embedded directly within each respective panel. Values in brackets represent IQRs. **(C)** Major-lineage patient-level deltas, expressed as a percentage of lymphocytes. Horizontal bars denote medians. **(D)** PD-1-excluded sensitivity screen using the same paired-test framework. PD-1-defined phenotypes represent assay-defined detectable staining rather than total receptor abundance. NK, natural killer; TNK, T and natural killer.

**Table 2:** Primary paired treatment-associated immune changes.

Feature	Direct parent denominator	Paired n	Pre median [IQR]	Post median [IQR]	Delta median [IQR], pp	Effect size	BH-FDR q
PD-1+ CD8 T cells	% of CD8+ T cells	20	21.00 [16.78, 27.80]	0.67 [0.23, 1.14]	-18.70 [-27.06, -13.66]	-0.990	1.4 x 10^-4
PD-1+ Vdelta1 gamma-delta T cells	% of Vdelta1 gamma-delta T cells	20	28.40 [18.00, 32.98]	4.34 [2.56, 8.59]	-17.81 [-24.23, -10.71]	-1.000	1.4 x 10^-4
PD-1+ Vdelta2 gamma-delta T cells	% of Vdelta2 gamma-delta T cells	20	10.06 [7.60, 14.98]	4.32 [2.69, 6.42]	-3.69 [-10.70, -1.82]	-0.857	0.005
CD19+ B cells	% of lymphocytes	20	11.05 [7.44, 14.03]	6.32 [4.84, 8.79]	-3.25 [-6.59, -1.89]	-0.962	3.3 x 10^-4

Values are medians [interquartile ranges]. Delta denotes post-treatment minus pre-treatment frequency in percentage points (pp). P-values were calculated using two-sided Wilcoxon signed-rank tests, and Benjamini-Hochberg false-discovery-rate correction was applied across all 75 prespecified immune phenotypes. Effect size is the paired rank-biserial correlation. PD-1-defined features represent assay-defined detectable staining after anti-PD-1 exposure. The complete 75-feature results are provided in **Supplementary Table S1**.

PD-1-defined results were interpreted with an assay caveat. PD-1+ CD8 T cells, PD-1+ Vδ1 γδ T cells, and PD-1+ Vδ2 γδ T cells changed after treatment, but these features reflect assay-defined detectable PD-1 staining under the study antibody-clone conditions. They should not be read as total PD-1 receptor expression or T-cell state without receptor-occupancy and clone-interference testing (24–31). Consistent with this epitope-masking hypothesis, the marked reduction in the assay-detected PD-1+ fractions of Vδ1 and Vδ2 γδ T cells mathematically necessitated a simultaneous relative enrichment of their complementary PD-1-negative fractions within these identical parent lineage gates. Indeed, the relative frequency of PD-1− Vδ1+ γδ T cells increased significantly across the paired cohort (median 71.60% to 95.66%; median delta +17.81 percentage points; P = 1.91×10^-^^6^), as did the frequency of PD-1− Vδ2+ γδ T cells (median 89.95% to 95.68%; median delta +3.69 percentage points; P = 2.61×10^-^^4^).

CD19+ B cells provided the principal non-PD1 anchor. In the primary paired screen, CD19+ B cells had a median post-pre change of -3.25 percentage points, paired effect size -0.962, and BH-FDR q=3.3e-04. In the PD-1-excluded sensitivity screen, CD19+ B cells remained q<0.05 (q=9.6e-04). This B-cell signal was interpreted descriptively in light of tumor-immunity and B-cell/tertiary lymphoid structure program (B/TLS) literature but was not assigned a mechanism (42–44).

### 3.3 Exploratory response-associated and clinical burden context

Response-associated immune analyses were exploratory. Across the 12-candidate response family and enriched response analyses, no response-stratified candidate reached BH-FDR q<0.05. **Figure 4** summarizes endpoint/context alignment, the MPR (including pCR) versus non-MPR delta-layer candidate screen, response-stratified trajectories and delta distributions, sensitivity estimates, baseline/burden context, and an exploratory statistical summary. All q>=0.05 response/pathology rows were visually de-emphasized.

**Figure 4:**
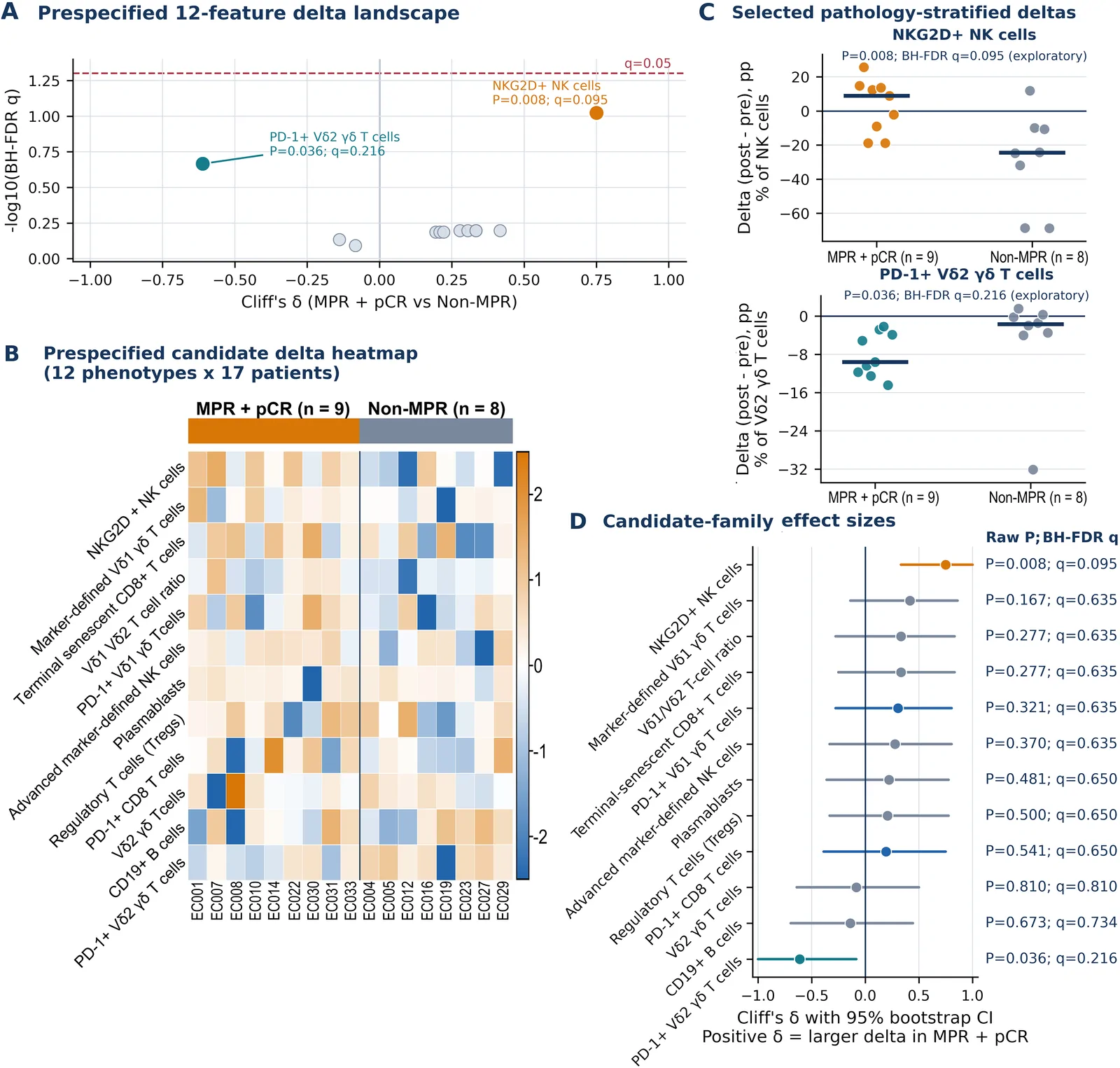
Exploratory pathology-stratified peripheral immune dynamics. Delta denotes post-treatment minus pre-treatment frequencies in percentage points (pp). **(A)** Effect-size landscape for the prespecified 12-candidate delta family. The dashed line denotes Benjamini-Hochberg false-discovery-rate (BH-FDR) q=0.05. **(B)** Heatmap of patient-level deltas, standardized within each phenotype. **(C)** Scatter plots of selected candidate deltas. Parent populations are explicitly defined on the y-axes, and group medians are indicated by horizontal bars. **(D)** Cliff’s δ effect sizes with 10,000-resample bootstrap 95% confidence intervals. Group comparisons used two-sided Mann-Whitney tests with BH-FDR correction. No candidate reached q<0.05; therefore, all pathology-associated findings are strictly exploratory. MPR, major pathologic response; pCR, pathologic complete response; NK, natural killer. Treg, regulatory T cells.

The most prominent pathology-stratified nominal pattern was NKG2D+ NK-cell delta. In the MPR (including pCR) group, the median delta was 8.9, compared with -24.5 in non-MPR, with median difference 33.4, Cliff’s delta 0.75, nominal P = 0.008, and BH-FDR q=0.095. PD-1+ Vδ2 γδ T-cell delta was also nominal only, with median difference -7.9, nominal P = 0.036, and BH-FDR q=0.216 (**Supplementary Table S2**). These rows are strictly exploratory and hypothesis-generating due to the small sample size and lack of significance after multiple-testing correction. They should be interpreted against NK-cell and γδ T-cell immunobiology without treating them as clinical decision tools (40, 41, 45, 46).

Lymph-node-positive ratio analyses provided clinical burden context. In the delta layer, q<0.05 rows were observed for PD-1+ Vδ2 γδ T cells (rho=0.713, q=0.023) and NKG2D+ NK cells (rho=-0.671, q=0.027) (**Supplementary Tables S3**, **S4**). These rows were not used to define a clinical decision rule. Baseline-dependence checks also produced q<0.05 rows for CD19+ B cells, PD-1+ Vδ2 γδ T cells, and NKG2D+ NK cells, indicating that baseline level may influence delta interpretation (**Supplementary Table S3**). Detailed exploratory response, clinical-burden, and baseline-dependence results are provided in **Supplementary Tables S2–S4**.

### 3.4 Public ESCC tissue transcriptomic contextualization

The public OMIX005710 analysis included 46 tissue samples from 22 patients (**Supplementary Figure S2**). In the treatment contrast (12 post-NAT versus 15 pre-NAT tumors), the B/TLS-like, NK cytotoxic/IFNG, and CD8 PDCD1/CXCL13 progenitor-like programs showed positive enrichment (NES = 1.618, 1.590, and 1.402; P = 0.027, 0.038, and 0.118; BH-FDR=0.100, 0.100, and 0.237, respectively). None reached BH-FDR<0.05. **Figure 1A** summarizes the GSEA landscape for the treatment and paired tumor-adjacent compartment contrasts, and **Figure 1B** shows the corresponding running-enrichment curves.

Across 27 tumor samples, **Figure 1C** shows correlations among the eight prespecified programs, and **Figure 1D** displays treatment-contrast leading-edge expression for the B/TLS-like, NK cytotoxic/IFNG, and CD8 PDCD1/CXCL13 progenitor-like programs. These tissue analyses provide biological context for the peripheral CD19+ B-cell, NKG2D+ NK-cell, and PD-1-defined CD8 T-cell findings. Complete GSEA, correlation, and leading-edge results are provided in **Supplementary Table S5**.

## 4 Discussion

This study defines longitudinal peripheral immune remodeling during anti-PD-1-based neoadjuvant therapy in ESCC. The primary paired analysis identified four FDR-supported treatment-associated phenotypes, with a reproducible CD19+ B-cell decrease as the principal non-PD-1 finding. A prespecified pathology-anchored analysis then identified nominal NK-cell and γδ T-cell delta patterns, while public tissue transcriptomics connected these peripheral axes to B/TLS, NK cytotoxic/IFNG, and PDCD1/CXCL13 progenitor-like programs.

The CD19+ B-cell finding is important because it remained detectable after excluding PD-1/CD279/PDCD1 features from the sensitivity screen. This non-PD1 anchor supports the interpretation that treatment-associated peripheral immune dynamics were not limited to PD-1 staining readouts. However, the result remains descriptive, and the biological significance of this observation remains uncertain and speculative. Crucially, because our analyses are based solely on relative cell frequencies rather than absolute cell counts, the observed reduction in CD19+ B-cell frequency should not be interpreted as a definite decrease in the absolute number of circulating B cells. Because systemic treatments, particularly cytotoxic chemotherapy, can profoundly alter total circulating leukocyte numbers through bone marrow suppression or immune cell redistribution to the tumor microenvironment, the observed proportional reduction in CD19+ B cells cannot definitively confirm a true numerical depletion. Furthermore, within a closed flow-cytometry parent gate, a decrease in the relative frequency of one specific subset mathematically necessitates a proportional increase in the complementary subset. This highlights a critical limitation of relying exclusively on relative frequencies. Prospective cohorts incorporating absolute lymphocyte counts, B-cell subset resolution, tissue B/TLS assessment, and spatial immune profiling would be needed to clarify its biological significance (42–44).

Contextualizing our findings within the broader literature of peripheral immune monitoring in ESCC reveals both consistencies and complementary perspectives. Previous studies investigating neoadjuvant anti-PD-1 therapy in ESCC have frequently reported systemic immune remodeling, typically characterized by significant alterations in peripheral T-cell subsets, such as shifts in CD4+/CD8+ ratios or regulatory T-cell frequencies (47, 48). While our data strongly align with this overarching theme of robust systemic immune modulation, our study distinctively highlights significant dynamics within non-T-cell lineages. Specifically, we observed a reproducible reduction in circulating CD19+ B cells, alongside an exploratory nominal association between NKG2D+ NK-cell dynamics and major pathologic response. Because the existing ESCC peripheral immunomonitoring literature is predominantly T-cell-centric, these findings provide a crucial complementary perspective. They suggest that broader innate and humoral immune compartments are also significantly mobilized or redistributed during neoadjuvant therapy, underscoring the need to expand future predictive biomarker investigations beyond conventional T-cell populations.

Because therapeutic anti-PD-1 antibodies mask detection epitopes via receptor occupancy, post-treatment decreases in detectable PD-1 staining may partly reflect steric hindrance or altered epitope availability rather than true receptor loss or altered T-cell function (24–31). Consequently, further biological interpretation of these PD-1-associated changes is strictly limited without orthogonal validation.

The exploratory response-associated analyses generated hypotheses but not outcome evidence. NKG2D+ NK-cell delta was the strongest nominal MPR-related pattern, and PD-1+ Vδ2 γδ T-cell delta was also nominal; neither reached BH-FDR q<0.05. Therefore, these findings should be consistently viewed as strictly exploratory and hypothesis-generating. These findings are compatible with NK-cell and γδ T-cell involvement in antitumor immunity but should not be used for response assignment or clinical decision-making, and they do not represent validated predictive biomarkers (40, 41, 45, 46). The lymph-node-positive ratio and baseline-depe NK cytotoxic/IFNG ndence layers add context by suggesting that immune deltas may relate to burden and starting immune state, but they do not constitute endpoint evidence.

The public tissue analysis extends the longitudinal blood findings into an independent ESCC tissue framework. Positive treatment-associated enrichment of B/TLS, and PDCD1/CXCL13 progenitor-like programs, together with their correlated variation across public tumors, provides a tumor-microenvironment setting in which the peripheral B-cell, NK-cell, and PD-1-defined T-cell observations can be interpreted. Because the cohorts are independent and the platforms measure different compartments, the tissue analysis provides biological context rather than patient-level validation (47, 49–51).

The principal limitations are the retrospective design, the 20-patient paired cohort and 17-patient pathology subset, attrition between baseline and post-treatment sampling, and heterogeneity in anti-PD-1 agents and chemotherapy backbones. Patients who completed paired sampling and surgery differ from patients without post-treatment samples. Absolute immune-cell counts, matched tumor tissue, and some run-level flow-cytometry metadata were unavailable. The independent public transcriptomic cohort cannot replace matched blood-tissue analysis. These considerations limit mechanistic extrapolation but do not alter within-patient description of treatment-associated immune-frequency changes.

The present data establish a longitudinal immune-pharmacodynamic framework rather than a single-time-point biomarker screen. Prospective studies should combine standardized sampling windows, absolute immune-cell counts, receptor-occupancy-aware PD-1 assessment, and matched blood-tissue profiling. Prospective studies should determine whether the reproducible CD19+ B-cell decrease and the exploratory NKG2D+ NK-cell and Vδ2 γδ T-cell trajectories correspond to defined cellular states and tissue programs. These findings support longitudinal peripheral immune monitoring during neoadjuvant therapy.

## 5 Conclusion

Anti-PD-1-based neoadjuvant therapy in ESCC was accompanied by broad, within-patient peripheral immune remodeling. The most robust non-PD-1 finding was a decrease in CD19+ B-cell frequency, while PD-1-defined CD8 and γδ T-cell phenotypes showed consistent changes in detectable staining. Pathology-anchored analyses identified exploratory NKG2D+ NK-cell and Vδ2 γδ T-cell delta patterns, and independent tissue transcriptomics placed the peripheral findings within B/TLS, NK cytotoxic/IFNG, and PDCD1/CXCL13 progenitor-like progenitor-like programs. Together, these results provide a longitudinal immune-pharmacodynamic framework for neoadjuvant therapy in ESCC.

## Data Availability

The datasets presented in this study can be found in online repositories. The names of the repository/repositories and accession number(s) can be found in the article/**Supplementary Material**.
