# Supplementary material for: Longitudinal peripheral immune dynamics during anti-programmed cell death 1 neoadjuvant therapy in esophageal squamous cell carcinoma
Source: Front Immunol. 2026 Oct 12;17:1917487. doi: 10.3389/fimmu.2026.1917487 (PMC13696451; doi:10.3389/fimmu.2026.1917487)
Supplement: Supplementary file 1 [file Table1.docx]

Supplementary Material

# 1 Supplementary Figures and Tables

## 1.1 Supplementary Figures

**Supplementary Figure 1. Representative flow cytometry gating strategy for peripheral immune subsets.** Lymphocytes were initially identified based on forward and side scatter characteristics (FSC-A vs. SSC-A). From the main lymphocyte gate, subsequent lineages were defined as follows: (1) Natural Killer (NK) cells (CD56+CD3−) and TNK cells (CD56+CD3+) were identified using a CD56 vs. CD3 plot. (2) B cells were identified by CD19+ expression following a CD45+ leukocyte pre-gate. (3) The overall T cell compartment was defined by CD3+ expression. Within the CD3+ gate, T cells were directly divided into parallel subsets: gamma-delta (γδ) T cells, CD4+ T cells, and CD8+ T cells. The γδ T cells were subsequently subdivided into Vδ1+ and Vδ2+ subsets. Both CD4+ and CD8+ T cells were further classified into naive (TNAIVE), central memory (TCM), effector memory (TEM), and terminally differentiated effector memory (TEMRA) subsets based on CD45RA and CCR7 expression. Within the CD4+ compartment, regulatory T cells (Tregs) were defined as CD25+CD127−. Additionally, CD4+ T cells were separated by CXCR5 expression into T follicular helper (Tfh, CXCR5+) and non-Tfh (T helper, CXCR5−) populations. These were further subdivided into Th1/Tfh1 and Th2/Tfh2 lineages utilizing CXCR3 and CCR4 markers, while Th17/Tfh17 lineages were identified utilizing CXCR3 and CCR6 markers.

**Supplementary Figure 2. Sample composition and predefined immune-program summaries in the public OMIX005710 ESCC tissue dataset.** (A) Distribution of 46 tissue samples from 22 patients by tissue compartment and treatment time point. Twelve post-NAT tumor samples and 12 post-NAT adjacent-tissue samples were available overall; 11 patients contributed matched tumor-adjacent pairs to the paired compartment analysis. (B) Coverage of genes in eight predefined tumor-microenvironment programs. (C) Median differences in program scores between post-NAT and pre-NAT tumor samples. (D) Median differences in selected cell-type proportions between post-NAT and pre-NAT tumor samples. NAT, neoadjuvant therapy; TME, tumor microenvironment.

## 1.2 Supplementary Tables Notes

**Supplementary Table S1. Full paired analysis of 75 peripheral immune phenotypes before and after neoadjuvant therapy.** The paired longitudinal cohort included 20 patients with immune measurements at both time points. Frequencies are reported relative to the feature-specific direct parent population. Delta was calculated as post-treatment minus pre-treatment and is expressed in percentage points (pp). Paired comparisons used two-sided Wilcoxon signed-rank tests, with Benjamini-Hochberg false-discovery-rate (BH-FDR) correction across all 75 phenotypes. Paired rank-biserial correlation is reported as the effect size.

**Supplementary Table S2. Associations between prespecified immune phenotypes and clinical or pathologic response.** Analyses included the pathology-evaluable paired cohort (MPR + pCR, n=9; non-MPR, n=8) and registry/radiographic response groups in the paired cohort (CR/PR, n=15; SD/PD, n=5). Baseline, post-treatment, and treatment-induced delta values were compared using two-sided Mann-Whitney U tests. Cliff's delta is reported as the between-group effect size, and BH-FDR correction was applied within each prespecified endpoint and analysis-layer family of 12 candidates.

**Supplementary Table S3. Association between baseline values and treatment-induced immune changes.** Baseline immune-phenotype values were correlated with treatment-induced delta values (post-treatment minus pre-treatment) among the 20 paired patients using Spearman rank correlations. BH-FDR correction was applied across the prespecified 12-feature family.

**Supplementary Table S4. PD-L1 combined positive score and associations with pathologic burden.** PD-L1 combined positive score (CPS) and residual tumor diameter are summarized among patients with available measurements. Associations between the 12 prespecified immune phenotypes and lymph-node-positive ratio were assessed at baseline, post-treatment, and delta layers using Spearman rank correlations. BH-FDR correction was applied within each 12-feature analysis layer; leave-one-out rho ranges are reported alongside the estimates.

**Supplementary Table S5. Public ESCC tissue** **pseudobulk, GSEA, and program correlations.** This multi-sheet workbook defines the independent OMIX005710 tissue cohort and analysis contrasts. It provides sample-level pseudobulk quality control, gene-set enrichment analysis (GSEA) results, leading-edge genes, program correlations, cell-type composition correlations, and sample-level module scores.

**Supplementary Table S6. Flow-cytometry antibody panel and gating definitions.** (A) Antibody-panel metadata used to identify the measured markers, including fluorochromes, clones, suppliers, and catalog numbers. (B) Phenotype dictionary defining each immune phenotype, its marker-based gating definition, direct parent denominator, reported unit, and PD-1 readout notes.

**Supplementary Table S7. Raw event counts for selected low-frequency peripheral immune-cell phenotypes.** This table reports the raw flow-cytometry event counts for 55 selected immune phenotypes across the 20 paired pre-treatment and post-treatment samples. Raw event counts were reviewed for low-frequency phenotypes; no subset-specific minimum-event threshold was applied, and zero/near-zero event observations were retained as recorded.
